# Host-Based Biomarkers in Saliva for the Diagnosis of Pulmonary Tuberculosis in Children: A Mini-Review

**DOI:** 10.3389/fped.2021.756043

**Published:** 2021-10-25

**Authors:** Nisreen Khambati, Laura Olbrich, Jerrold Ellner, Padmini Salgame, Rinn Song, Else Margreet Bijker

**Affiliations:** ^1^Oxford Vaccine Group, Department of Paediatrics, University of Oxford, Oxford, United Kingdom; ^2^Division of Infectious Diseases and Tropical Medicine, University Hospital, Ludwig Maximilian University of Munich, Munich, Germany; ^3^German Center for Infection Research (DZIF), Partner Site Munich, Munich, Germany; ^4^Department of Medicine, New Jersey Medical School, Rutgers, The State University of New Jersey, Newark, NJ, United States; ^5^Division of Infectious Diseases, Boston Children's Hospital, Boston, MA, United States

**Keywords:** saliva, tuberculosis, biomarker, diagnosis, host-specificity

## Abstract

The diagnosis of pulmonary tuberculosis (TB) in children remains a significant challenge due to its paucibacillary nature, non-specificity of symptoms and suboptimal sensitivity of available diagnostic methods. In young children particularly, it is difficult to obtain high-quality sputum specimens for testing, with this group the least likely to be diagnosed, while most at risk of severe disease. The World Health Organization (WHO) has prioritized research into rapid biomarker-based tests for TB using easily obtainable non-sputum samples, such as saliva. However, the role of biomarkers in saliva for diagnosing TB in children has not been fully explored. In this mini-review, we discuss the value of saliva as a diagnostic specimen in children given its ready availability and non-invasive nature of collection, and review the literature on the use of host-based biomarkers in saliva for diagnosing active pulmonary TB in adults and children. Based on available data from adult studies, we highlight that combinations of cytokines and other proteins show promise in reaching WHO-endorsed target product profiles for new TB triage tests. Given the lack of pediatric research on host biomarkers in saliva and the differing immune response to TB infection between children and adults, we recommend that pediatric studies are now performed to discover and validate salivary host biosignatures for diagnosing pulmonary TB in children. Future directions for pediatric saliva studies are discussed, with suggestions for technologies that can be applied for salivary biomarker discovery and point-of-care test development.

## Introduction: The Challenges of Pediatric TB Diagnosis

In 2019, there were 192,000 deaths in children due to tuberculosis (TB), although most experts agree this figure is underestimated (1). Over 90% of child TB deaths occurred in those not receiving treatment, predominantly due to difficulties in diagnosis (2). The majority of missed TB cases occurs in children under five, the group with the highest risk of severe disease (3). Diagnosis of pulmonary TB in children is challenging due to its paucibacillary nature, non-specific symptoms, and limitations of the available diagnostic tests (4–6). Sputum microscopy is positive in <15% of children with probable TB and culture has a long turn-around time (7). Molecular DNA-based methods such as GeneXpert^®^ are faster but require a stable electricity supply, limiting useability in low resource settings (8). Moreover, these tests require high-quality respiratory samples, which is problematic in young children who cannot expectorate sputum on demand, necessitating collection of gastric aspirates, nasopharyngeal aspirates, or induced sputum (9). These procedures are invasive and require trained staff (10), potentially missing children not presenting to secondary healthcare. In the absence of microbiological confirmation, diagnostic scoring systems based on clinical and radiological information are often used with variability in performance (7), and the risk of misdiagnosis (11).

The development of non-sputum-based point-of-care (POC) tests on easily obtainable samples like blood, urine, stool, and saliva has been prioritized by the World Health Organization (WHO) (12). These diagnostic approaches ideally should be feasible in primary care settings with the purpose of starting therapy during the same clinical encounter (13). Specific target product profiles (TPP) relating to the performance of new tests in children have been outlined by the WHO, with minimal targets of 66% sensitivity and 98% specificity for a diagnostic test and 90% sensitivity and 70% specificity for a triage test (12).

Diagnostic tests based on biomarkers have gained attention due to their potential for translation into non-sputum POC technologies (14). Biomarkers are characteristics that objectively indicate a normal biological or pathogenic process (15). Biomarker tests can be host-based, measuring the immune response following infection (16), or pathogen-based, identifying components of *Mycobacterium tuberculosis* (Mtb) (17). They may be able to identify culture-negative TB in early stages (18), which could help target the high number of children with microbiologically unconfirmed TB.

The role of saliva as a diagnostic specimen for pulmonary TB in children has not been fully explored. Detection of pathogen DNA using GeneXpert MTB/RIF in saliva from adults demonstrated low sensitivity (39%) compared to sputum (19), and sensitivity in children is likely to be lower due to the paucibacillary nature of pediatric disease. Moreover, the fact that TB disease in young children is often more severe and more frequently disseminated than in adults (20) conceivably makes children more suited to host-based diagnostics. This review therefore focuses on host-based salivary biomarkers. We outline the evidence on host biomarkers in saliva for diagnosing active pulmonary TB in relation to the WHO-endorsed TPP. We demonstrate the research gaps in children and based on available data from adult studies, discuss the potential for salivary biomarkers for children with recommendations for future pediatric studies.

## Saliva as a Diagnostic Specimen in Children

Saliva contains various components enabling its potential as a sample for diagnosing infectious diseases (21). For example, detection of specific IgM in saliva has been used to confirm infection with measles, mumps and rubella (22). Congenital cytomegalovirus infection in neonates can be diagnosed through detection of viral DNA in saliva (21), and there has been interest in diagnosing SARS-CoV-2 infection in children using saliva instead of nasopharyngeal swabs (23). There is also a growing body of pediatric research demonstrating the clinical utility of salivary biomarkers for predicting metabolic syndrome (24) and diagnosing and monitoring chronic conditions like chronic kidney disease (25, 26) and hypertension (27). Whilst salivary assay development for adult conditions has increased, diagnostics specifically for pediatrics remain comparatively limited (21).

Saliva offers specific advantages compared to other sample types. It can be collected non-invasively and painlessly which is attractive for children and allows for repeated sampling (28). Importantly for TB, collection does not require highly skilled personnel, enabling useability in primary healthcare settings (29). As a mucosal and airway associated specimen, saliva may be better for studying host biomarkers in pulmonary TB compared to blood (30). Subbian et al. found that gene expression signatures in lung biopsies from TB patients only partly corresponded to those identified in their blood (31), suggesting that the lung-specific host response is not fully captured in blood-based biomarkers (32). With the first pathogen-host contact in pulmonary TB occurring in the oral and nasal passages, saliva may more accurately represent the immune response in the respiratory system (33).

Various collection systems are available for children. Whole saliva collected through passive drool into a collection tube is recognized as the optimal method (34). This avoids the potential issue of absorbent material interfering with analytes or the volume of saliva collected (35). It also may allow for a more consistent sample by avoiding saliva collection from specific salivary glands (36). However, this technique is only possible for older children who can cooperate (37). For infants and young children, absorbent oral swabs or eye spears which have a cellulose sponge on a shaft that can be inserted into the mouth, are feasible and safe (38, 39). Saliva is extracted from the absorbent device through centrifugation or compression (34).

## Evidence for Saliva Biomarkers in Pulmonary TB Diagnosis

### Materials and Methods

We conducted a search to identify studies which reported the diagnostic performance of salivary host-based biomarkers for active pulmonary TB. PubMed and Web of Science databases were searched for diagnostic studies published up to 8th August 2021 using the search text words: “saliva” and “(tuberculos^*^ OR TB OR Mtb)” and associated medical subject headings: “saliva,” “mycobacterium tuberculosis,” and “tuberculosis.” Animal studies were excluded. We did not specifically include “children” as a search term in line with the Cochrane Handbook for Systematic Reviews of Diagnostic Test Accuracy (40) to maximize sensitivity of the search. We found nine studies on host salivary diagnostic biomarkers, published between 1973 and 2021. Since only one study involved children (41), we also reviewed the evidence from eight adult studies (30, 42–48). Supplementary Figure 1 outlines the search procedure and Supplementary Table 1 summarizes the included studies.

### TB-Specific Antibodies in Saliva

A study in the USA in 1973 evaluated levels of hemagglutinating IgA to tuberculoprotein and tuberculopolysaccharide in saliva (47). Salivary antibodies were detected in only one of 20 TB cases, and also in one of 20 COPD patients, indicating very poor sensitivity and specificity. Later studies found more promising results. Araujo et al. evaluated secretory IgA in saliva against the 38 kDa antigen of Mtb in Venezuelan children using ELISA (41). Based on 34 children with TB and 46 healthy controls, sensitivity was only 36%, but specificity was 91%. Raras et al. also evaluated secretory IgA levels, although they measured antibodies against a recombinant semi-purified 38 kDa antigen in saliva from 30 TB adult cases and 30 healthy controls in Indonesia using dot blot (42). In contrast to Araujo et al., sensitivity was higher at 80%, but specificity was lower at 37% (42). Differences in population, geography and type of antibody assay may account for some of the variation between the two studies. The reference standard also differed: while Raras et al. defined a positive TB case based on sputum smear positivity (i.e., high bacillary burden) (42), Araujo et al. relied on clinical, radiological or microbiological information with only 3 of 34 positive cases confirmed bacteriologically (41).

Anti-38 kDa antibodies in blood generally offer good specificity but their low sensitivity has limited their potential as serological tests in diagnosing TB (49). Measurement in saliva does not appear to perform any better. In both studies of anti-38 kDa antibodies in saliva, diagnostic performance fell considerably short of TPP criteria. Moreover, both studies used a case control design instead of including a spectrum of patients in whom the test would be applied in clinical settings. This approach can overestimate diagnostic performance (50). In summary, despite their potential for translation into simple assays (16), the data on TB-specific antibodies in saliva as stand-alone tests for diagnostic purposes have not been encouraging to date.

### Cytokines and Other Inflammatory Proteins as Biomarkers

Five studies measured salivary levels of selected cytokines, growth factors, enzymes and acute phase proteins in adults with confirmed TB or symptoms of TB using Luminex multiplex assays (30, 43–46). Of the 54 biomarkers analyzed in more than one study, eight showed statistically significant differences (*p* < 0.05) between TB patients and negative controls, albeit only one study reported correction for multiple comparisons (46). These eight biomarkers included alpha 2 macroglobulin (A2M), serum amyloid P (SAP), interferon gamma-induced protein (IP-10), vascular endothelial growth factor (VEGF), interleukin 6 (IL-6), monocyte chemoattractant protein-1 (MCP-1), fraktaline, and c-reactive protein (CRP). Sensitivities and specificities of these individual biomarkers ranged between 36 and 83% and between 52 and 96%, respectively (Table 1). Crucially, six biomarkers showed opposing trends between studies. For example, IP-10 was higher in TB patients in one study (46) whereas it was higher in healthy controls in another (45). Only salivary CRP and fraktaline showed a similar pattern across studies and were raised in TB cases in more than one study. No individual biomarkers met TPP criteria for a diagnostic or triage test.

**Table 1:** Summary of individual biomarkers analyzed in more than one study using Luminex bead-based multiplex assays.

	**First author of study (sample size)**				
	**Phalane (** **43** **) (38)**	**Jacobs (** **44** **) (51)**	**Jacobs (** **45** **) (104)**	**Namuganga (** **30** **) (78)**	**Estevez (** **46** **) (70)**
A2M		Sensitivity 50% Specificity 88%	Sensitivity 75% Specificity 71%		
Haptoglobin			Sensitivity 88% Specificity 81%		
CRP	Sensitivity 46% Specificity 93%		Sensitivity 75% Specificity 85%		
SAP		Sensitivity 83% Specificity 52%	Sensitivity 72% Specificity 72%		
PCT					
Ferritin					
TPA					
Fibrinogen			Sensitivity 63% Specificity 75%		
SAA					
sFas					
Granzyme A					
sFasl					
sCD137					
BCA-1					
IFN-y					
IFN-A2					Sensitivity 71% Specificity 66%
IP-10			Sensitivity 72% Specificity 53%		Sensitivity 81% Specificity 64%
MIP-1A					Sensitivity 96% Specificity 43%
MIP-1B	Sensitivity 46% Specificity 93%				
TNF-A					
TNF-B					
VEGF			Sensitivity 63% Specificity 74%		Sensitivity 75% Specificity 67%
sCD40L					
MMP-2					
MMP-9					
IL-1A					Sensitivity 81% Specificity 65%
IL-1B		Sensitivity 39% Specificity 97%			
IL-2					
IL-4					
IL-5	Sensitivity 27% Specificity 96%				
IL-6	Sensitivity 64% Specificity 81%		Sensitivity 63% Specificity 71%		Sensitivity 65% Specificity 72%
IL-7					
IL-8			Sensitivity 53% Specificity 78%		
IL-9	Sensitivity 27% Specificity 96%				
IL-10					
IL-12(p-70)					
IL-12(p-40)					
IL-13					
IL-15					
IL-16		Sensitivity 50% Specificity 85%			
IL-17A		Sensitivity 72% Specificity 67%			
IL-17F					
IL-21					
IL-22					
IL-23		Sensitivity 61% Specificity 85%			
IL-33					
Fraktaline	Sensitivity 36% Specificity 96%				Sensitivity 79% Specificity 81%
GM-GSF					
EGF					
MCP-1			Sensitivity 53% Specificity 67%		Sensitivity 60% Specificity 82%
TGF-A					Sensitivity 57% Specificity 94%
GCSF					
GRO					Sensitivity 71% Specificity 79%
MDC					

*Biomarker not analyzed in study*.

*
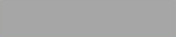
 No significant difference in biomarker between TB cases and negative controls or biomarker not detected/barely detected in saliva*.

*
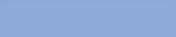
 Levels of biomarker significantly higher in TB cases compared to negative controls*.

*
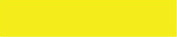
 Levels of biomarker significantly lower in TB cases compared to negative controls*.

*Biomarkers with statistically significant differences (*p* < 0.05) in concentration between TB cases and negative populations are shaded in blue and yellow and presented with their corresponding diagnostic accuracy estimates*.

Compared to individual biomarkers, combinations of markers in biosignatures showed more potential. Table 2 summarizes the best performing biosignatures from each study. The most promising biosignatures originated from a prospective cohort study in South Africa with a low risk of bias by Jacobs and colleagues (44). They recruited 51 adults based on symptoms of pulmonary TB and compared biomarker levels between those with culture positive TB and with other respiratory diseases in a blinded manner. An eight-marker biosignature involving salivary granzyme A, growth differentiation factor 15 (GDF-15), serum amyloid A (SAA), epithelial-neutrophil activating peptide 78 (ENA-78), IL-12(p40), IL-13, IL-21, and plasminogen activator inhibitor-1 (PAI-1) had a sensitivity of 93% and specificity of 100% (40). This biosignature met TPP for both a triage and diagnostic test. Another eight-marker biosignature involving extracellular matrix protein 1 (ECM1), myoglobulin, hemofiltrate CC chemokine 1 (HCC1), tissue plasminogen activator (TPA), ENA-78, IL-12 (p40), IL-13, and IL-21 had a sensitivity of 100% and a specificity of 95%, reaching TPP for a triage test (44). Both these biosignatures were defined in adults who were HIV negative. The optimal biosignature evaluated in both HIV positive and negative individuals, included IL-1β, IL-23, ECM-1, HCC1, and fibrinogen, and had a sensitivity of 89% and specificity of 90% (44). Biosignatures from the other Luminex studies performed less well, but were overall closer in approaching the WHO-endorsed TPP than individual biomarkers (Table 2).

**Table 2:** Optimal biosignatures in relation to WHO-endorsed target product profiles for triage tests (90% sensitivity and 70% specificity) and diagnostic tests (66% sensitivity and 98% specificity).

**References**	**Assay**	**Optimal biosignature (no. of biomarkers included)**	**AUC (95% CI)**	**Sensitivity % (95% CI)**	**Specificity % (95% CI)**	**Meets minimal TPP for triage tests**	**Meets minimal TPP for diagnostic tests**
Phalane et al. (43)	Luminex	IL-5, IL-6, IL-15, TNF-α, and CRP (5)	NR	82 (NR)	81 (NR)	No	No
Jacobs et al. (44)	Luminex	IL-1β, IL-23, ECM-1, HCC1, and fibrinogen (5)	0.88 (0.77–0.99)	89 (77–100)	90 (60–97)	No	No
		Granzyme A, GDF-15, SAA, IL-21, ENA-78, IL-12(p40), IL-13, and PAI-1 (8)	0.99 (0.98–1.0)	93 (77–100)	100 (75–100)	Yes	Yes
		ECM1, myoglobulin, HCC1, IL-21, ENA-78, TPA, IL-12 (p40), and IL-13 (8)	NR	100 (83–100)	95 (68–100)	Yes	No
Jacobs et al. (45)	Luminex	CRP, ferritin, SAP, MCP-1, A2M, fibrinogen, and TPA (7)	NR	78 (60–90)	83 (72–91)	No	No
Namuganga et al. (30)	Luminex	G-CSF, TNF-α, and VEGF (3)	NR	42 (NR)	75 (NR)	No	No
Estevez et al. (46)	Luminex	Fractalkine, IP-10, IL-1α, and VEGF (4)	0.88 (NR)	74 (NR)	91 (NR)	No	No
Mutavhatsindi et al. (48)	LC–MS/MS	AACT, NAD(P)H-hydrate epimerase, PSMB6, IGKV1-33, and neuroserpin (5)	1.0 (1.0–1.0)	100 (76–100)	91 (59–100)	Yes	No
		Flavin reductase, myosin- 9, neuroserpin, and protein S100-A11 (4)	NR	91 (NR)	91 (NR)	Yes	No

*Diagnostic accuracies were investigated by receiver operator characteristics curve analysis with models using logistic regression or general discriminant analysis to identify the optimal combination of markers for active TB diagnosis*.

*AUC, area under the curve; CI, confidence intervals; LC-MS/MS, liquid chromatography with tandem mass spectrometry; NR, not recorded; TPP, Target Product Profiles; TB, Tuberculosis*.

There was little overlap in the optimal biosignatures identified across the Luminex studies. Only tumor necrosis factor α (TNF-α), CRP, fibrinogen, and VEGF appeared in the optimal biosignatures from more than one study. There are various possible explanations for this heterogeneity. All studies except one (46) reported cross validation methods to reduce overfitting of the data in their models. However, they did not validate results in an independent cohort to demonstrate generalizability. The diagnostic thresholds to define sensitivity and specificity for each biomarker and the statistical methods to determine these thresholds also varied amongst the studies. In terms of population, studies recruited from different geographic regions. Host genetics, microbiome, and coinfections may therefore vary, also attributing to heterogeneity in host response (32). Many of the promising biomarkers were acute phase reactants which could be affected by infections other than TB and otherwise altered immune states (43). Apart from HIV status, no study included information on other diseases that could have influenced biomarker levels. Lastly, two studies included healthy individuals as their negative controls (43, 46). This could overestimate diagnostic performance compared to those using clinically relevant negative populations (30, 44, 45).

### Shotgun Proteomics

In a discovery study, Mutavhatsindi et al. performed label-free liquid chromatography with tandem mass spectrometry on saliva from 22 adults with symptoms of TB (48). Following correction for multiple testing, they identified 26 differentially expressed proteins between adults with culture-positive TB and those with other respiratory diseases. Five proteins diagnosed TB with an area under the ROC curve above 0.8 (macrophage-capping protein, plasminogen, profilin-1, f-actin-capping protein subunit beta, and alpha-1-antichymotrysin). Most of these 26 proteins have not previously been investigated in this context, however, many are involved in inflammation, activation of the immune system and enzyme regulation (48). Similarly to the Luminex studies described above, TPP criteria were only achieved when markers were used in combination. After leave-one out cross validation, a five protein biosignature diagnosed TB with a sensitivity of 100% and specificity of 90.9% and a four protein biosignature showed a sensitivity and specificity of 90.9% (Table 2). Whilst validation in larger studies is necessary, these results further demonstrate the potential of multiple salivary proteins as diagnostic biomarkers.

## Discussion: Potential in Children and Future Directions

We reviewed the current evidence on salivary host-based biomarkers for diagnosing pulmonary TB. Based on adult studies, combinations of cytokines and other proteins appear more promising than single proteins or antibodies. Despite the heterogeneity, many of these proteins are involved in the inflammatory response and may be elevated in diseases other than TB (51), therefore, specificity may be an issue in saliva host-based tests. This is important in children in whom respiratory infections are common. Saliva host diagnostics therefore primarily have potential as triage tests, distinguishing between those unlikely to have TB and those requiring confirmatory testing. Triage tests have a lower specificity requirement (>70% minimally) compared to diagnostic tests (98%) (12), but they need a high sensitivity and negative predictive value given the high fatality rate in young children. Ahmad and colleagues found that addition of serum antibodies against the TB antigen Ag85B to a four-cytokine blood-based triage test increased sensitivity from 80 to 86% and specificity from 65 to 69% (52). Although this fell just short of TPP criteria, it illustrates a useful strategy for enhancing diagnostic performance by expansion of protein-based tests with another diagnostic assay.

With the exception of one study on immunoglobulins in children, all studies on salivary biomarkers were conducted in adults. Despite the lack of pediatric research, salivary proteins in children are worth exploring. Host cytokine biosignatures in blood have showed promise in reaching TPP for triage tests in both adults (53) and children (54), although the blood cytokine expression profile differed between the two populations. Disappointing salivary cytokine biomarkers from adult studies do not necessary preclude value for children. Given the complex differences in the TB immune response between children and adults (32, 55), candidate salivary biomarkers for diagnostic assays may vary. Therefore, early phase pediatric studies should first be targeted to identify biosignatures which can identify symptomatic children with microbiologically confirmed TB (56).

Various technologies exist for biomarker discovery in saliva. High-throughput multiplex assays with bead-based (Luminex) or planar (Mesoscale) technology can measure multiple proteins simultaneously, with minimal sample requirements compared to traditional methods like ELISA (57). However, such platforms rely on targeted antibody capture-based strategies and issues relating to antibody specificity and cross-reactivity can limit the number of multiplexable targets (58). Shotgun proteomics, although more expensive and resource-intensive, may be a more comprehensive and unbiased method to biomarker discovery (48). A proteomics approach also has potential to discover novel proteins within extracellular vesicles, such as exosomes, which are found in saliva and have shown promise as non-invasive diagnostic markers for other pulmonary diseases (59, 60).

Subsequently, biosignatures identified from discovery studies should be independently validated in well-characterized cohorts where the test is clinically indicated (51). These cohorts should include children with the full spectrum of suspected TB and with other respiratory diseases presenting similarly to TB (56). Further validation in multiple populations under real-life field conditions will further assess robustness and accuracy and inform selection of biomarkers for potential implementation into POC tests (61). Conducting studies in diverse geographical locations will also help capture how genetic heterogeneity and location-specific exposure to microorganisms impact upon host responses to TB (32).

Measurement of several biomarkers using multiplex immunoassays or shotgun proteomics is not feasible at lower levels of care. However, recent developments have highlighted the possibility of translating validated biosignatures into affordable POC technology. Application of a lateral flow assay detecting two blood chemokines was feasible in a multi-center study in Africa, without requiring a cold chain for storage or distribution (62). Three- and six-biomarker POC tests using fingerstick blood are currently being validated as triage tests at multiple African sites (63). Whilst the viscosity of saliva and potentially low concentration of analytes present specific challenges for saliva-based POC platforms, electrochemical immunosensors have been suggested as possible solutions (64). Such biosensors contain an electrochemical transducer and have been used successfully to detect cytokines in saliva at low detection limits (65). Unique advantages, including high sensitivity, low cost, simple operation, rapid response and functionality in turbid media (64, 66) mark them as potential tools for future saliva POC diagnostic devices.

Future pediatric studies should prioritize recruitment of those under five (67). Given that pediatric TB is heterogeneous, approval of diagnostics for children under five should ideally be based on data specifically from this age group. Collecting clinical information on comorbidities, particularly HIV, malnutrition and respiratory coinfections, would help appreciate how disease phenotype can influence the host response. Unlike in adults, sputum-based reference tests are often not appropriate in young children. Microbiological reference standards based on combinations of alternative samples such as gastric or nasopharyngeal aspirates are therefore recommended (68). To ensure comparability of results across studies, classification of children should conform to the National Institutes of Health consensus definitions for childhood pulmonary tuberculosis (i.e., confirmed, unconfirmed, and unlikely TB) (69). Finally, the significant upfront investment in setting up clinical pediatric cohorts has led to recommendations to establish pediatric specimen biorepositories (67). As improved diagnostics become available, storage of saliva may allow future biomarker testing from cohorts with different geographical locations, age groups, and comorbidities.

## Conclusion

Saliva could be a valuable specimen for testing host biomarkers to diagnose pulmonary TB in children, however very little research in this population exists. Discovery studies in adults show that host salivary proteins, when used in combination as biosignatures, demonstrate considerable promise as triage tests. Given the differing TB immune response in children, studies in pediatric populations are now needed. The ready availability of saliva and non-invasive nature of collection is appealing for children, particularly those under five. Late inclusion of children into clinical studies of new technologies is a major barrier to progress in childhood TB (3). The COVID-19 pandemic is expected to have worsened TB control, with 6.3 additional million cases estimated to occur between 2020 and 2025, and children are especially vulnerable (1). Novel and optimized diagnostics in children are now more than ever urgently required.

## Author Contributions

NK, RS, and EB devised the review and the main conceptual ideas. NK drafted the manuscript. LO, JE, PS, RS, and EB critically revised the work. All authors approved the final version of the manuscript.

## Funding

Research reported in this publication was supported by the National Institute of Allergy and Infectious Diseases of the National Institutes of Health under Award Number R01AI152159. The content is solely the responsibility of the authors and does not necessarily represent the official views of the National Institutes of Health.

## Conflict of Interest

The authors declare that the research was conducted in the absence of any commercial or financial relationships that could be construed as a potential conflict of interest.

## Publisher's Note

All claims expressed in this article are solely those of the authors and do not necessarily represent those of their affiliated organizations, or those of the publisher, the editors and the reviewers. Any product that may be evaluated in this article, or claim that may be made by its manufacturer, is not guaranteed or endorsed by the publisher.

## Abbreviations

A2M: alpha 2 macroglobulin
AACT: alpha-1- antichymotrypsin
AUC: area under the curve
BCA-1: B cell-attracting chemokine 1
CI: confidence intervals
CRP: c-reactive protein
EGF: epidermal growth factor
ENA-78: epithelial-neutrophil activating peptide 78
ECM1: extracellular matrix protein 1
GCSF: granulocyte-colony stimulating factor
GM-CSF: granulocyte-macrophage colony-stimulating factor
GDF-15: growth differentiation factor 15
HCC1: hemofiltrate CC chemokine 1
IFN-y: interferon gamma
IFN-A2: interferon alpha-2
IP-10: interferon gamma-induced protein 10
IL-1A: interleukin 1 alpha
IL-1B: interleukin 1 beta
IL-2: interleukin 2
IL-4: interleukin 4
IL-5: interleukin 5
IL-6: interleukin 6
IL-7: interleukin 7
IL-8: interleukin 8
IL-9: interleukin 9
IL-10: interleukin 10
IL-12(p-40): interleukin 12(p-40)
IL-12(p-70): interleukin 12(p-70)
IL-13: interleukin 13
IL-15: interleukin 15
IL-16: interleukin 16
IL-17A: interleukin 17A
IL-17F: interleukin17F
IL-21: interleukin 21
IL-22: interleukin 22
IL-23: interleukin 23
IL-33: interleukin 33
IGKV1-33: immunoglobulin kappa variable 1–33
LC-MS/MS: liquid chromatography with tandem mass spectrometry
MDC: macrophage-derived chemokine
MIP-1A: macrophage inflammatory protein 1-alpha
MIP-1B: macrophage inflammatory protein 1-beta
MMP-2: matrix metalloproteinase-2
MMP-9: matrix metalloproteinase-9
MCP-1: monocyte chemoattractant protein-1
MGIT: Mycobacteria growth indicator tube
Mtb: Mycobacterium tuberculosis
NR: not recorded
PCT: procalcitonin
PAI-1: plasminogen activator inhibitor-1
PSMB6: proteasome subunit beta type-6
SAA: serum amyloid A
SAP: serum amyloid P component
sCF40L: soluble CD40 ligand
sCD137: soluble CD137
sFAS: soluble Fas
sFASl: soluble Fas ligand
SD: standard deviation
TPA: tissue plasminogen activator
TB: Tuberculosis
TPP: Target Product Profile
TGF-A: transforming growth factor-alpha
TST: tuberculin skin test
TNF-A: tumor necrosis factor alpha
TNF-B: tumor necrosis factor beta
VEGF: vascular endothelial growth factor
WHO: World Health Organization
ZN: Ziehl Neelson.
