# Supplementary material for: Host-Based Biomarkers in Saliva for the Diagnosis of Pulmonary Tuberculosis in Children: A Mini-Review
Source: Front Pediatr. 2021 Oct 25;9:756043. doi: 10.3389/fped.2021.756043 (PMC8575443; doi:10.3389/fped.2021.756043)
Supplement: Supplementary file 1 [file Data_Sheet_1.docx]

# Supplementary Figure 1: Flow diagram showing procedure for searching studies and reasons for study exclusion from mini-review

# Mtb: Mycobacterium tuberculosis

Articles identified through database searching

PubMed: (n=191)

Web of Science: (n=171)

Reasons for exclusion (n=18)

Pathogen-based biomarker: (n=10)

No data on diagnostic accuracy: (n=2)

Review: (n=2)

Duplicate: (n=1)

Not saliva data: (n=1)

Full text not available: (n=1)

Not related to Mtb: (n=1)

Full text articles assessed following screening of title/abstract (n=27)

Studies included in mini review

(n=9)

# Supplementary Table One: Summary of the nine published studies on host-based biomarkers in saliva for diagnosing active TB

* Proteins refer to cytokines, growth factors, enzymes, acute phase proteins, transcription regulators and others but exclude antibodies.

**All microbiological confirmatory tests for the positive reference standard were done on sputum samples

LC-MS/MS: liquid chromatography with tandem mass spectrometry; MGIT: Mycobacteria growth indicator tube; NR: not recorded; SD: standard deviation; TST: tuberculin skin test; TB; Tuberculosis; ZN: Ziehl-Neelson

| **First Author** | **Year Published** | **Site** | **Study design** | **Sample size (n)** | **Mean age (in years) ±SD** | **No. (%) with HIV** | **Type of biomarker** | **Assay to detect biomarker** | **Positive reference standard** (n)** | **Negative control (n)** |
| --- | --- | --- | --- | --- | --- | --- | --- | --- | --- | --- |
| *Barlow^47^* | 1973 | USA | Case control | 40 | NR | NR | Antibody | Hemagglutination | Microbiological confirmation- method NR (20) | Hospitalized patients with COPD and no evidence of TB (20) |
| *Araujo^41^* | 2004 | Venezuela | Case control | 80 | 8 ± 5 | 0 | Antibody | ELISA | Composite standard based on clinical, radiological and microbiological information- smear microscopy or culture (34) | Healthy contacts from endemic country (46) |
| *Phalane^43^* | 2013 | South Africa | Case control | 38 | 38± 10 | 8 (21.0) | Proteins* | Luminex | Culture -MGIT (11) | Healthy individuals from endemic country (27) |
| *Raras^42^* | 2014 | Indonesia | Case control | 60 | >17± NR | NR | Antibody | Dot blot | Smear microscopy -ZN staining (30) | Healthy individuals from endemic country (30) |
| *Jacobs^44^* | 2016 | South Africa | Cohort | 51 | 36 ± 10 | 12 (23.5) | Proteins* | Luminex | Culture -MGIT (18) | Other respiratory disease (33) |
| *Jacobs^45^* | 2016 | South Africa | Cohort | 104 | 39 ± 12 | 18 (17.3) | Proteins* | Luminex | Composite standard based on clinical, radiological and microbiological information- MGIT (32) | Other respiratory disease (72) |
| *Namuganga^30^* | 2017 | Uganda | Cross sectional | 78 | 32± 14 | 13 (16.7) | Proteins* | Luminex | Culture- MGIT (39) | Other respiratory disease (39) |
| *Estevez^46^* | 2020 | Spain | Case control | 70 | 40± NR | NR | Proteins* | Luminex | Culture and/or nucleic acid amplification (28) | Healthy contacts with no TB infection (42) |
| *Mutavhatsindi ^48^* | 2021 | South Africa | Cohort | 22 | 39 ± 9 | 0 | Proteins* | LC–MS/MS | Culture- MGIT (11) | Other respiratory disease (11) |
